# Effects of tranexamic acid on platelet function and thrombin generation (ETAPlaT): WOMAN trial sub-study

**DOI:** 10.12688/wellcomeopenres.9964.1

**Published:** 2016-12-15

**Authors:** Kastriot Dallaku, Haleema Shakur, Ian Roberts, Phil Edwards, Danielle Beaumont, Maria Delius, Braun Siegmund, Orion Gliozheni, Ilir Tasha, Saimir Cenameri, Ulrich Mansmann

**Affiliations:** 1 Institute for Medical Information Sciences, Biometry and Epidemiology, Ludwig Maximilian University of Munich, Munich, Germany; 2 University Hospital of Obstetrics Gynecology “Koço Gliozheni”, Tirana, Albania; 3 Clinical Trials Unit, London School of Hygiene & Tropical Medicine, London, UK; 4 Department of Obstetrics and Gynaecology, Ludwig Maximilian University of Munich, Munich, Germany; 5 German Heart Centre, Technical University of Munich, Munich, Germany

**Keywords:** Postpartum Hemorrhage, Tranexamic Acid, Coagulation Factors, Platelet function

## Abstract

**Background**. Postpartum haemorrhage (PPH) is a leading cause of maternal death. Tranexamic acid (TXA) has the potential to reduce bleeding and a large randomized placebo controlled trial of its effect in women with PPH (The WOMAN trial) is underway. TXA might also affect coagulation factors and platelets. **Objectives**. To examine the effect of TXA on thrombin generation, platelet function, fibrinogen, D-dimer and coagulation factors in women with PPH. **Methods**. We will conduct a sub-study within the WOMAN trial. Women with clinically diagnosed primary PPH after vaginal or caesarean delivery are eligible for inclusion. Blood samples will be collected at baseline and 30 minutes after the first dose of study treatment. Using platelet poor plasma we will measure thrombin generation, fibrinogen, D-dimer, factor V and VIII, and Von Willebrand factor. Platelet function will be evaluated in whole blood using Multiplate® tests. **Outcomes**. The primary outcome is the effect of TXA on thrombin generation. Secondary outcomes include the effect of TXA on platelet function, fibrinogen, D-dimer and coagulation factors.

## Abbreviations

ADPtest: Test of Adenosine Di Phosphate; AUC: Area Under Curve; CI: Confidence Interval; CBC: Complete Blood Count; DMC: Data Monitoring Committee; ETP: Endogenous Thrombin Potential; FV: Coagulation Factor V; FVIII: Coagulation Factor VIII; ICH GCP: International Conference on Harmonisation of Good Clinical Practice; LT: Lag Time; LSHTM: London School of Hygeine and Tropical Medicine; PH: Peak Height; MPV: Mean Platelet Volume; PPH: postpartum haemorrhage; SAP: Statistical Analysis Plan; SOP: Standard Operating Procedure; TtP: time to peak; TGA: Thrombin Generation Assay; TXA: Tranexamic Acid; TRAPtest: Test of Thrombin Receptor of Thrombocyte; TCC: Trial Coordinating Centre; TSC: Trial Steering Committee; vWF: Coagulation Factor Von Willebrand.

## Background

Tranexamic acid (TXA) reduces bleeding by inhibiting the enzymatic degradation of fibrin blood clots by the serine protease plasmin. During fibrinolysis, plasminogen binds to fibrin via lysing binding sites, where it is converted to the active fibrinolytic enzyme plasmin by tissue plasminogen activator. TXA is a molecular analogue of lysine that inhibits fibrinolysis by reducing the binding of plasminogen to fibrin. TXA reduces surgical bleeding and in 2010, the CRASH-2 trial showed that TXA reduces death due to bleeding in trauma patients ( Roberts & Prieto-Merino, 2014). TXA also has the potential to reduce death due to bleeding in PPH. The WOMAN trial is investigating the effect of TXA on mortality and morbidity in women with PPH.

In addition to its role in fibrinolysis, plasmin has various effects on coagulation factors. Coagulation factors V (FV) and VIII (FVIII) are first activated and then inactivated by plasmin. The initial activation of these factors may generate enough thrombin to have a pro-coagulant effect. Plasmin might also promote thrombin generation by inactivating tissue factor pathway inhibitor, a major inhibitor of tissue factor-mediated thrombin generation ( Godier *et al.*, 2012). Ogiwara *et al.* (2010) showed that thrombin generation is increased in the presence of plasmin. If plasmin increases thrombin production, TXA, by inhibiting plasmin production, should reduce thrombin generation.

Although TXA reduces bleeding by inhibiting fibrinolysis, it may reduce bleeding through other mechanisms. For example, TXA may improve platelet function ( Boylan *et al.*, 1996). The exposure of platelets with plasmin induces platelet activation ( Ervin & Peerschke, 2001). Plasmin activates platelets via different mechanisms, such as stimulating the arachidonic acid cascade, and inducing platelet degranulation and complement activation ( Godier *et al*., 2012). The main effect of TXA on platelet function may be due to the inhibition of plasmin, and consequently the decrease of plasmin’s multifactorial induction on platelets.

TXA improved platelet function in cardiac surgery patients, with impaired platelet function caused by preoperative exposure of antiplatelet therapy, such as clopidogrel ( Shi *et al.*, 2013). Another study in cardiac surgery patients ( Weber *et al*., 2011) noticed that administration of TXA significantly improved platelet function by attenuating platelet aggregation defects created by antiplatelet treatment.

In patients with chronic renal failure, administration of TXA corrected platelet aggregation defects ( Mezzano *et al*., 1999), and as result improved platelet function. Also, Sabovic *et al.* (2005) reported that platelet dysfunction in haemodialysis patients was effectively corrected with long term administration of TXA at a low dose.

## Methods and study design

### Objective

The study will assess the effect of TXA on thrombin generation, coagulation factors and platelet function in women with PPH, in particular, the effects of TXA on thrombin generation, factor V (FV), factor VIII (FVIII), Von Willebrand factor (vWF), fibrinogen, D-dimers and platelet function. We hypothesize that by inhibiting plasmin, TXA will decrease thrombin generation, and modify platelet activity, FV, FVIII and vWF levels, in patients with PPH.

### Trial design

The WOMAN-ETAPlaT is a sub-study of the World Maternal Antifibrinolytic Trial, an international randomized, double blinded, placebo-controlled trial. As a sub-study, there are no changes to the study design of the WOMAN trial, but for the specific design of the sub-study there are some additional examinations and laboratory tests.

***Participants and eligibility criteria.*** *Inclusion criteria*: Participants in the study are women ≥18 years old with a clinical diagnosis of postpartum haemorrhage (PPH) after vaginal or caesarean birth. All available treatment for PPH should be given, and inclusion in the study should be considered as soon as possible after patient consent has been obtained. Clinically diagnosed PPH can be established with one of the following conditions: amount of blood lost following vaginal birth is >500mL or >1,000mL after caesarean birth; or enough blood loss has compromised the patient’s hemodynamic status. *Exclusion criteria*: Patients diagnosed with PPH, for whom the physician believes that there is a secure indication or contraindication for use of TXA, should not be randomised.

***Study setting.*** For the conduct of this sub-study, a single site has been chosen, the Obstetric Gynaecology University Hospital ”Koço Gliozheni” in Tirana, Albania. This is justified because PPH is the main cause of maternal mortality in Albania. The hospital offers tertiary health care and is a national referral hospital for other obstetric gynaecology hospitals in the country.

***Interventions.*** Patients with clinically diagnosed PPH and fulfilling the eligibility of the WOMAN trial will be randomized into the study after the administration of the standard treatments for PPH. As soon as primary PPH is diagnosed, the study drug (TXA or placebo) will be administered as soon as possible alongside all other clinically indicated treatments for PPH. Each trial treatment pack contains two doses, and each dose contains two ampoules of 500mg TXA or placebo (sodium chloride 0.9%).

### Outcomes

***Measures of the primary and secondary outcomes of ETAPlaT study.*** The primary outcome will be the effect of TXA on thrombin generation [thrombin generation assay (TGA) parameter – endogenous thrombin potential (ETP)]. Secondary outcomes will include the effect of TXA on platelet function (Multiplate® tests: ADPtest and TRAPtest), fibrinogen, D-dimer and FV, FVIII, vWF levels, and other TGA parameters [lag time (LT); time to peak (TtP); and peak height (Ph)]. Levels of all parameters will be assessed on two venous blood samples, collected at the baseline and between 30±15 minutes after the first dose of study treatment is given.

Further secondary outcomes will include assessment of the relationship between these parameters, patient demographics and clinical data (in the time frame from randomization to the patient discharge from the hospital or until 42 days).

***Outcomes of the main study WOMAN trial.*** The WOMAN trial will not provide information about platelet function, thrombin generation, fibrinogen, D-dimers and coagulation factors V, VIII and vWF in patients receiving TXA/placebo. However, the most reliable estimates of the effects of TXA on the outcomes for women with PPH will be provided by the WOMAN trial.

### Sample size

We aim to assess if TXA, when compared to a placebo, has an effect on change over time on the TGA parameter, ETP. Therefore, the sample size calculation is based on this parameter. Patients are expected to be equally allocated between the two intervention groups; 88 patients per group (total, 176 patients) are needed to detect the difference of ETP 243nM/min, with a change between the groups at a 5% significance level with a power of 80%.

### Randomization and blinding

Patients with a clinical diagnosis of PPH, who are eligible for the WOMAN trial, after having completed the informed consent procedures, will be randomly assigned to receive either TXA or placebo. Patients randomised in the trial will receive the study drug (TXA/placebo) by intravenous injection. The lowest available number of the pack will be used first and then subsequently other packs from the box that contains 8 study treatment packs. The blinding process will be carried out by an independent supplier, who will mark the study drug with the blinded label. The vials and packages will be the same, and the randomisation number will be used as the pack identification.

### Procedures before/after the study treatment administration, blood sample collection and analysis

**Figure 1.**
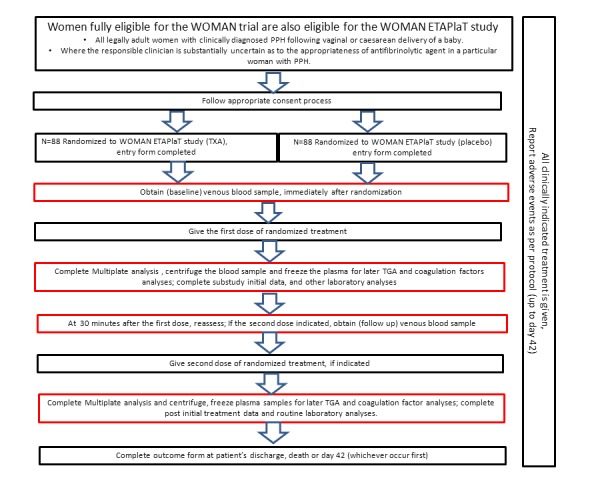
Algorithm of the WOMAN-ETAPlaTsub-study. Black boxes: Standard procedure of the WOMAN trial. Red boxes: Additional procedures required for the ETAPlaT sub-study.

Immediately after patient is randomized, two test tubes (3mL and 5mL) of venous blood will be taken and the first dose of trial treatment will be administered. About 30±15 minutes after the administration of the first dose of trial drugs, two more test tubes (3mL and 5mL) of venous blood will be collected. If a woman continues to bleed and a second dose of trial treatment is required, the second blood sample will be taken before this is given ( Figure 1).

Samples of the 3mL tubes (hirudine) whole blood will be analyzed immediately at the hospital in Tirana, Albania for platelet function using a Multiplate Platelet Function Analyzer (Dynabyte GmbH, Munich, Germany).

Samples of 5mL tubes (sodium citrate 3.2%) will be centrifuged immediately at 3000xg for 20 minutes, and platelet poor plasma samples will be divided ito two aliquots and preserved at -80°C. These will be transferred to dry ice for later analysis at the Institute of Laboratory Medicine, German Heart Centre in Munich, Germany for TGA, fibrinogen, D-dimers, and FV, FVIII and vWF levels. The temperature storage conditions of the plasma samples will be monitored and recorded daily.

As part of the routine protocol of the hospital, a blood sample for complete blood count (CBC) is taken from every woman in labour prior to delivery. In cases of PPH randomized in the study, another CBC is performed after 4 hours and within 12 hours, depending on the severity of PPH. The pre-post randomization difference in CBC parameters, such as haemoglobin or haematocrit drop, will be evaluated.

Standard operating procedures for handling, storage and analysis of the blood samples and the process for transferring the data to the Trial Coordinating Centre (TCC) are developed by each responsible Institution, and are approved by TCC. Copies are available from the Trial Master File.

### Ethical considerations, recruitment and consent

Ethical approval for WOMAN ETAPlaT protocols was obtained 28.10.2013 (ref. 6518) from the London School of Hygiene and Tropical Medicine (LSHTM) Ethics Committee in London, United Kingdom, and by the National Ethics Committee in Tirana, Albania on 11.07.2013 (ref. 62) and amendment on 01.12.2014 (ref. 81). PPH is an obstetric emergency and it is not possible to predict which women that will have this complication. In this emergency situation it is important to carry out an informed consent procedure, according to regulatory requirements, and adhere to the ICH-GCP and Declaration of Helsinki, for patients to be eligible for the study. The consent procedure will be performed as per the approved procedure for the WOMAN trial.

Briefly, advanced information about the trial will be given to pregnant women, which will make the recruitment and consent procedure easier. The consent process will depend on the clinical emergency and the patient’s physical or emotional state. If the woman is fully competent, the healthcare giver will provide information and discuss with her about the study and obtain informed written consent ( Supplementary File 1 and Supplementary File 2). If the woman’s mental or physical capacity does not allow her to give consent, the woman’s relative or representative, if available, should be informed about the trial and informed written consent will be obtained by them ( Supplementary File 1 and Supplementary File 3). Where no relatives or representative are available, a waiver of prior written consent has been approved for the WOMAN trial. Where a waiver of prior written consent is used, the woman, relative or representative should be asked for consent for the continuation of the trial procedure.

To minimize the need for multiple information sheets and consent forms, one form, which combines the WOMAN trial and the WOMAN-ETAPlaT study, will be used.

## Data collection and monitoring

### Data collection

All study data will be gathered on the WOMAN trial entry data form (baseline information) and outcome data form (patient death, discharge from the hospital or 42 days after randomization). Additional information will be collected with ETAPlaT data collection forms ( Supplementary File 4). All study data will be sent electronically to the main database of the TCC in London, UK. ETAPlaT data collection forms will include the following:

- Time when the blood sample was taken and laboratory analysis was started and completed, and any technical problems with analysis;
- Laboratory results of Multiplate analysis (ADPtest, TRAPtest), TGA, D-dimer, fibrinogen, F V, FVIII and vWF;
- Treatment given that may affect coagulation;
- Other data about the woman’s parity, BMI, gestational age at birth, maternal pre-existing conditions (anaemia, cardiac disease, haemoglobinopathy, chronic hypertension, renal disease, treatment or prophylaxis with antithrombotic drugs, or history of previous thromboembolism), pregnancy related conditions (preeclampsia, diabetes, infection, placental abruption), labour induction/augmentation and duration, anaesthesia (epidural, spinal or general), birth weight, additional doses of uterotonics (oxytocin/methergine).

### Analysis

Analysis will compare two groups of randomized patients, allocated in TXA group or placebo group on an intention-to-treat basis, regardless of treatment received, and outcomes will be presented as appropriate effect estimates and 95% confidence intervals.

In analysis of primary outcome, TXA and placebo groups will be compared regarding the TGA parameter, ETP of baseline and follow up values. The same analysis will be performed for secondary outcomes such as platelet function (ADPtest and TRAPtest), coagulation factors (FV; FVIII; vWF, Fibrinogen and D-Dimer) and other TGA parameters (LT, TtP, and Ph). A subgroup analysis for primary outcome will be based on main risk factors for PPH, such as uterine atony, placental factors and genital trauma.

A detailed statistical analysis plan (SAP) will be finalized prior to the sub-study unblinding for final analysis. All statistical analysis will be conducted with R and STATA statistical package.

### Limitations of the study

One limitation of this sub-study is the small number of the patients that will be recruited. Another potential limitation is withdrawal of participation by patients from this study, or risk of loss of contact in case of complications after discharge from the hospital and within the period of 42 days after the birth.

### Trial Steering Committee (TSC) and Data Monitoring Committee (DSC)

A TSC for the WOMAN trial is in place and agreed to the conduct of this sub-study. Decisions of the TSC can influence immediately on the continuation of the WOMAN‐ETAPlaT study. Information about the WOMAN‐ETAPlaT study will be reported routinely to the TSC by Haleema Shakur. An independent DMC is already in place for the WOMAN trial. Adverse events, which occur during the study period and are related directly to the WOMAN‐ETAPlaT study, will be reported to the DMC.

The sub-study had its responsibilities coordinated by the TCC, which may attribute responsibilities to third parties, and will be detailed by suitable arrangements.

### Trial status

Recruitment for the WOMAN ETAPlaT sub-study started on November 2013 and the final participant follow-up was completed in March 2015. Data cleaning is ongoing.

## Supplementary files

**Supplementary File 1:** Information sheet for patient and their representative (Albanian and English).

**Supplementary File 2:** Patient consent form (Albanian and English).

**Supplementary File 3:** Representative consent form (Albanian and English).

**Supplementary File 4:** WOMAN-ETAPlaT data collection forms.
