# Supplementary material for: Effects of tranexamic acid on platelet function and thrombin generation (ETAPlaT): WOMAN trial sub-study
Source: Wellcome Open Res. 2016 Dec 15;1:29. [Version 1] doi: 10.12688/wellcomeopenres.9964.1 (PMC5234699; doi:10.12688/wellcomeopenres.9964.1)
Supplement: Supplementary file 1 [file wellcomeopenres-1-10739-s0000.tgz › a29c9cf8-1e44-4dac-bfd1-c47854a930bd.docx]

## Information sheet for patient and her representative (Albanian)

Dr. Kastriot Dallaku, Spitali Universitar Obstetrik Gjinekologjik Koço Gliozheni,

Blv. Bajram Curri, Tirane, AlbaniaTel. +355 6920 54212, Email: kastriotdallaku@yahoo.com

FLETË INFORMATIVE PËR PACIENTEN E PËRFAQËSUESIN (IT) E SAJ

**(Studimi PËR FEMRAT) WOMAN TRIAL dhe WOMAN-ETAPlaT**

**TITULLI I STUDIMIT**:

(1) Acidi traneksamik për trajtimin e hemorragjisë së paslindjes: Një studim ndërkombëtar, i randomisuar, dyfish i verber, placebo dhe i kontrolluar.

(2) WOMAN-ETAPlaT – Efekti i Acidit Tranexamik ne funksionin trombocitar dhe gjenerimin e trombines, ne nje pjese te pacienteve te Studimit per FEMRAT.

NUMRI I QENDRES SE STUDIMIT: 001

**VERSIONI I FLETËPALOSJES:** VERSIONI 1.1 DATA: 3 qershor 2013

**Ky spital po merr pjesë në një studim kërkimor ndërkombëtar, për të gjetur mënyra për të përmirësuar trajtimin e femrave që kanë gjakrrjedhje të rëndë pas lindjes së fëmijës.**

1. **Ne dëshirojmë t’ju ftojmë që të merrni pjesë në këtë studim.**
2. **Në këtë studim jeni përfshirë kur keni qenë në një gjendje shumë të rëndë dhe ne dëshirojmë që të vazhdoni të merrni pjesë.**
3. **Si përfaqësues i pacientes, ju kërkojmë që të merrni një vendim në emër të saj.**

*(Ju lutemi të rrethoni atë zgjedhje që është e aplikueshme)*

**Mjeku i studimit tashmë ka kontrolluar për të siguruar që ju/pacientja jeni e përshtatshme nga ana mjekësore për këtë hulumtim dhe ju kërkohet që të merrni një vendim, nëse ju/pacientja mund të përfshiheni në këtë studim.** Kjo fletushkë ju jep të dhëna për këtë studim, duke përfshirë këtu arsyet se përse po bëhet ky studim, si dhe rreziqet dhe përfitimet e pjesëmarrjes në të.

JU LUTEMI TË LEXONI ME KUJDES INFORMACIONIN E SHËNUAR MË POSHTË DHE DREJTOJINI MJEKUT OSE MAMISË QË KUJDESET PËR JU, NDONJË PYETJE QË MUND TË KENI.

**1) Cili është qëllimi i këtij studimi?**

Në këtë spital, gratë që kanë gjakrrjedhje shumë të rëndë pas lindjes së fëmijës (gjithashtu njihet me emrin **hemorragji e paslindjes**) marrin trajtimet e disponueshme më të mira. Qëllimi i këtij studimi kërkimor është që të shikojë nëse ka ndonjë trajtim më të mirë për femrat që kanë gjakrrjedhje të rëndë pas lindjes së fëmijës. Ne shpresojmë që trajtimi (**acidi traneksamik**) do të ndihmojë gjakun që të mpikset më shpejt, dhe në këtë mënyrë të pakësojë sasinë e gjakut të humbur dhe të zvogëlojë nevojën për transfuzion gjaku dhe trajtime të tjera.

Por është gjithashtu e mundur që trajtimi në këtë studim të krijojë mpiksje gjaku aty ku nuk është e nevojshme dhe meqenëse ilaçi nuk përdoret rregullisht pas lindjes së fëmijës, ne akoma nuk i dimë të gjitha efektet anësore të mundshme. Ne shpresojmë që të arrijmë të zbulojmë që trajtimi do të ketë më tepër të mira sesa dëme, por akoma nuk e dimë këtë gjë.

**2) Përse po bëhet ky hulumtim?**

Hemorragjia e paslindjes mund të jetë një gjendje shëndetësore mjaft e rëndë dhe ngandonjëherë për të kontrolluar gjakrrjedhjen është e nevojshme të bëhet operacion. Me mijëra femra në mbarë botën vdesin çdo vit nga kjo sëmundje dhe është e rëndësishme të gjenden mënyra më të mira për të kontrolluar gjakrrjedhjen e tepërt pas lindjes së fëmijës.

Acidi traneksamik shpeshherë përdoret për të ulur gjakrrjedhjen pas operacioneve të mëdha si p.sh., operacionet e zemrës. Disa femra që kanë gjakrredhje të rëndë të menstruacioneve (perioda) përdorin gjithashtu acidin traneksamik. Ky studim për FEMRAT po zhvillohet për të parë nëse acidi traneksamik mund të ulë gjakrrjedhjen tek femrat që kanë gjakrrjedhje pas lindjes.

**3) Përse jeni përfshirë ju?**

Mjeku juaj ju ka diagnostikuar me hemorragji të paslindjes. Mjeku juaj ka kontrolluar që jeni e përshtatshme për këtë studim, por është në dorën tuaj nëse vendosni të merrni pjesë apo jo.

**4) Kush po e bën këtë studim dhe kë mund të telefononi nëse keni ndonjë pyetje ose ndonjë problem?**

Dr Kastriot Dallaku është mjeku përgjegjës për këtë studim në këtë spital, ne bashkepunim me grupin kryesor te studimit: Profesor Orion Gliozheni, Dr Ilir Tasha dhe Dr Saimir Cenameri. Ky studim koordinohet nga mjekë dhe një grup studimi në Shkolla Shendetsis dhe Mjekësise Tropikale në Londër (Universiteti i Londrës). Nëse keni ndonjë pyetje mund të kontaktoni me mjekun tek:

| Adresa | Spitali Universitar Obstetrik Gjinekologjik Koço Gliozheni, Tirane, Shqiperi |
| --- | --- |
| Telefoni | +355 6920 54212 |

Mund të vizitoni lirisht faqen e internetit të këtij eksperimenti, për të qenë në dijeni për zhvillimin e mëtejshëm të këtij eksperimenti: www.thewomantrial.Lshtm.ac.uk

**5) Një paciente nuk mund të jetë në këtë studim, nëse:**

- Mjeku mendon se ka një arsye të caktuar se përse acidi traneksamik **nuk duhet** dhënë aspak.
- Mjeku mendon se ka një arsye të caktuar se përse **duhet** dhënë acidi traneksamik.
- Pacientja nuk është në moshë të rritur.

**6) Çfarë do të ndodhë/ka ndodhur gjatë këtij studimi?**

Do t’ju jepen të gjitha trajtimet e zakonshme në raste urgjence për gjakrrjedhje të rëndë pas lindjes së fëmijës, duke përfshirë këtu dhe lëngje për të zëvendësuar gjakun që keni humbur. Do t’ju jepet gjithashtu ose një dozë acidi traneksamik ose placebo (solucion fiziologjik, që nuk përmban acid traneksamik). Kjo dozë do t’ju jepet me anë të një injeksioni në venë. Nëse pas rreth 30 minutash keni akoma gjakrrjedhje, ose nëse gjakrrjedhja ndalon dhe fillon përsëri brenda 24 orëve pas dozës së parë, mund t’ju jepet një dozë e dytë e ngjashme. Nuk do t’ju bëhen më tepër se dy injeksione për këtë studim.

Juve do tju merren dy mostra gjaku per analiza nga venat nepermjet nje age. Mostra e pare do te merret para se tju administrohet mjekimi i studimit, dhe mostra e dyte e gjakut do tju merret tridhjete deri gjashtedhjete minuta me vone. Ju nuk do te paguani per analizat qe do te kryhen nga gjaku I ketyre dy mostrave.

Ne nuk e dimë nëse dhënia e acidit traneksamik pas marrjes së të gjithë trajtimeve të tjera do të ndihmojë apo jo, kështu që gjysma e femrave në këtë studim do të marrin acid traneksamik dhe gjysma tjetër do të marrë një bar inaktiv. Zgjedhja se cilin trajtim do të merrni, bëhet tërësisht me zgjedhje të rastësishme dhe do të keni një mundësi të barabartë për të marrë ndonjërën prej tyre. As ju, as mjeku që ju mjekon nuk do të dini se cilin trajtim do të merrni. Ky informacion mbahet në një listë mirëbesimi në një vendndodhje të pavarur në Londër. Studimi nuk përfshin teste të mëtejshme, por mjeku/mamia juaj do të dërgojë disa detaje të shkurtra për trajtimin dhe shërimin tuaj pranë Qendrës së Koordinimit në Londër. Ata do të dërgojnë gjithashtu disa informacione për shëndetin e bebes/bebeve tuaja. Nëse pas daljes nga spitali dhe deri në 42 ditë pas trajtimit do t’ju shfaqet ndonjë problem mjekësor, ju lutemi të lajmëroni mjekun emrin e të cilit e gjeni të shënuar në këtë formë. Ky informacion do të përdoret në mirëbesim të plotë nga njerëzit që punojnë në këtë studim dhe nuk do të shpërndahet aspak në asnjë lloj rrethane.

**7) Cilat janë rreziqet e mundshme të pjesëmarrjes në këtë studim?**

Acidi traneksamik NUK është një ilaç i ri dhe përdoret gjerësisht për të ulur gjakrrjedhjen në gjendje të tilla shëndetësore si p.sh., në kirurgjinë madhore te zemrës. Nuk ka asnjë provë vendimtare të ndonjë efekti të rëndë anësor me përdorim të shkurtër kohor. Por trajtimi i studimit mund të shkaktojë mpiksje të gjakut aty ku nuk nevojitet dhe meqenëse ilaçi nuk përdoret rregullisht pas lindjes së fëmijës, ne nuk i dimë të gjitha efektet anësore të mundshme. Mjeku juaj do t’u raportojë organizatorëve të këtij eksperimenti për ndonjë problem të paparashikuar që mund t’u dalë.

**8) Cilat janë përfitimet e mundshme të pjesëmarrjes në këtë studim?**

Ne shpresojmë që acidi traneksamik do të mund të ndihmojë për të ulur humbjen e gjakut. Njohuritë që fitojmë nga ky studim do të ndihmojë në të ardhmen femrat me hemorragji të paslindjes në mbarë botën.

**9) Çfarë të dhënash mbajmë në mënyrë private?**

Të gjithë informacionin për ju, si dhe shkaku i gjakrrjedhjes pas lindjes së fëmijës, do të mbahen në mënyrë private. Të vetmit njerëz që do të lejohen t’i shikojnë të dhënat do të jenë mjekët që po drejtojnë këtë studim, punonjësit në Qendrën e Koordinimit dhe organet rregulluese që kontrollojnë që studimi po zhvillohet ashtu siç duhet. Qendra e Koordinimit të Eksperimentit mund të dojë të marrë ose kopjojë disa dokumenta të eksperimentit, të cilat do të kenë emrin tuaj dhe këtu do të përfshihet Formulari i Miratimit i nënshkruar. Kjo do t’i ndihmojë ata që të sigurojnë se ky eksperiment po zhvillohet ashtu siç duhet. Të dhënat tuaja do të mbeten në mirëbesim dhe do të mbahen në një vend të sigurtë magazinimi në Qendrën e Koordinimit të Eksperimentit.

Të dhënat tuaja në mirëbesim do të mbahen të ndara më vete nga të dhënat e eksperimentit dhe do të shkatërrohen brenda pesë vitesh pas mbarimit të eksperimentit. Ne do t’i publikojmë rezultatet e studimit në një revistë mjekësore, në mënyrë që mjekë të tjerë të përfitojnë nga kjo njohuri, por të dhënat tuaja personale NUK do të përfshihen dhe nuk do të ketë asnjë mënyrë ku ju mund të identifikoheni.

**10) A mund të ndërroni mendje për pjesëmarrjen në studim?**

Mund të tërhiqeni kur të doni nga ky studim në çfarëdo lloj kohe. Duhet të thoni për shembull*“Kam vendosur që nuk dua të jem më në këtë studim”*. Ne shpresojmë që do të na lejoni t’i përdorim të dhënat se si kaluat, por nëse nuk dëshironi që ne t’i përdorim, ju lutemi t’i thoni mjekut.

**11) Ç’gjë tjetër ju nevojitet të dini?**

- Në ato raste kur diçka nuk shkon siç duhet dhe lëndoheni gjatë këtij studimi, Shkolla për Higjenën dhe Mjekësinë Tropikale në Londër që po organizon këtë studim do të jetë përgjegjëse për ato kërkesa që kanë të bëjnë me dëmet jo-neglizhuese që kanë rrjedhur si rezultat i pjesëmarrjes në këtë studim.
- Ne do t’ju kërkojmë të nënshkruani më vete një formular miratimi dhe do t’ju japim të mbani një kopje të këtij formulari. Mund ta mbanigjithashtukëtëfletushkë me informacione.
- Ky studim është rishikuar dhe miratuar nga Komiteti i Etikës Studimore.

**12) Çfarë ndodh më pas?**

Nëse pas daljes nga spitali dhe deri në 42 ditë pas lindjes së fëmijës do t’ju shfaqet ndonjë problem, ne patjetër që duam të dimë për të. Do t’ju jepet një kartë me detajet e kontaktit të mjekut të studimit në këtë spital, të cilën duhet ta mbani në një vend të sigurtë dhe t’ua tregoni kujtdo që mund t’ju mjekojë për ndonjë sëmundje.

Nëse dëshironi të keni një kopje të rezultateve përfundimtare të këtij studimi, ju lutem të lajmëroni mjekun e studimit dhe ai/ajo do të sigurojë që të merrni një kopje kur të publikohet.

## Information sheet for patient and her representative (English)

Dr. Kastriot Dallaku, Obstetric Gynecology University Hospital Koço Gliozheni,

Blv. Bajram Curri, Tirane, Albania; Tel. +355 6920 54212, Email: kastriotdallaku@yahoo.com

INFORMATION SHEET FOR THE PATIENT AND HER REPRESENTATIVE(S)

**THE WOMAN TRIAL and WOMAN-ETAPlaT**

**TITLE OF RESEARCH**:

1. Tranexamic acid for the treatment of postpartum haemorrhage: An international randomised, double blind, placebo controlled trial.

2. WOMAN-ETAPlaT – Effect of Tranexamic Acid on Platelet Function and Thrombin Generation, in a sample of participants of WOMAN trial

**TRIAL SITE NUMBER:** 001

**LEAFLET VERSION:** 1.1 dated 03 June 2013

**This hospital is taking part in an international research study to find ways to improve the treatment of women who have severe bleeding after delivery of their baby.**

1. **We would like to invite you to take part in this study**
2. **When you were very unwell you were included in this study and we would like you to continue to take part**
3. **As a representative of the patient we are asking you to make a decision on her behalf***(Please circle the option that applies)*

**The Research Doctor has already checked to make sure you/the patient is medically suitable for this research and you are being asked to make a decision about whether you/the patient can be included in this study.** This sheet gives information about the study, including the reasons why the study is being done, and the risks and benefits of taking part.

PLEASE READ THE INFORMATION BELOW CAREFULLY AND ASK THE DOCTOR OR MIDWIFE LOOKING AFTER YOU ANY QUESTIONS YOU MAY HAVE.

**1) What is the purpose of the study?**

In this hospital, women who have a very severe bleeding after childbirth (also called **postpartum haemorrhage**) are given the best available treatments. The aim of this research study is to see if there is a better treatment for women who have severe bleeding after childbirth. We hope that the treatment (**tranexamic acid**) will help the blood to clot sooner, and so lessen the amount of blood lost and reduce the need for a blood transfusion and other treatments. But it is also possible that the study treatment may cause clots where they are not needed, and because the drug is not routinely used after childbirth, we do not know all the likely side effects. We hope to find that the treatment will do a little more good than harm but we don’t yet know this.

**2) Why is this research being done?**

Postpartum haemorrhage can be a very serious condition and sometimes requires surgery to control the bleeding. Many thousands of women worldwide die each year from this condition and it is important to find better ways of controlling excessive bleeding after childbirth.

Tranexamic acid is often used to reduce bleeding after major operations such as heart operations. Some women who have heavy menstrual bleeding (periods) also use tranexamic acid. The WOMAN study is being done to see if TXA can reduce bleeding in women with postpartum bleeding.

**3) Why have you been invited?**

You have been diagnosed with postpartum haemorrhage by your doctor. Your doctor has checked that you are suitable for the study, but it is up to you whether or not you decide to take part.

**4) Who is doing the study and who can you call if you have any questions or problems?**

Dr Kastriot Dallaku is in charge of this study at this hospital, together with the study team: Prof Orion Gliozheni, Dr. Ilir Tasha, Dr. Saimir Cenameri. The study is coordinated by doctors and a trial team at The London School of Hygiene & Tropical Medicine (University of London). If you have any questions you can contact the doctor at:

| Address: | Obstetric Gynaecology University Hospital Koço Gliozheni, Tirana, Albania |
| --- | --- |
| Telephone: | +355 6920 54212 |

You are also free to visit the trial website to keep up to date with the progress of the trial: www.thewomantrial.Lshtm.ac.uk

**5) A patient cannot be in this study if:**

- The doctor thinks there is a particular reason why tranexamic acid definitely **should not** be given
- The doctor thinks there is a particular reason why tranexamic acid definitely **should** be given
- They are not an adult

**6) What will happen/has happened during this study?**

You will be given all the usual emergency treatments for severe bleeding after childbirth, including fluids to replace the blood that you have lost. You will also be given a dose of either the tranexamic acid or a placebo (a liquid which doesn’t contain tranexamic acid). This dose will be given as an injection into your vein. If after about 30 minutes you are still bleeding, or if the bleeding stops and starts again within 24 hours after the first dose, you may be given a second dose of the same. You will not receive more than two injections for the study.

You will also have two blood samples for analysis taken from your vein through a needle. The first sample will be taken before you are given any of the trial treatment, and the second sample thirty to sixty minutes later. You will not have to pay for any of the tests done on these two blood samples.

We do not know whether giving tranexamic acid on top of all the other treatments will help or not, so half the women in the study will receive tranexamic acid and the other half will receive a placebo. The choice of which treatment you receive is completely random and you will have an equal chance of receiving either one. Neither you nor the doctor treating you will know which treatment you receive. This information is kept on a confidential list at an independent location in London. The study involves no extra tests but your doctor/midwife will send brief details about your treatment and recovery to the Coordinating Centre in London. They will also send information about the health of your baby/ies. If after discharge from hospital and up to 42 days after treatment you develop any medical problems, please let the doctor named on this form know. This information will be used in strict confidence by the people working on the study and will not be released under any circumstances.

**7) What are the possible risks of being in the study?**

Tranexamic acid is NOT a new drug and it is widely used to reduce bleeding in conditions such as major heart surgery. There is no conclusive evidence of serious side effects with short term use. But the study treatment may cause clots where they are not needed and, because the drug is not routinely used after childbirth, we do not know all the likely side effects. Your doctor will report to the trial organisers any unexpected problems you may have.

**8) What are the possible benefits of being in the study?**

We hope that tranexamic acid may help reduce blood loss. The knowledge that we gain from this study will help women with postpartum haemorrhage worldwide in the future.

**9) What information do we keep private?**

All information about you and the reason for bleeding after childbirth will be kept private. The only people allowed to look at the information will be the doctors who are running the study, the staff at the Coordinating Centre and the regulatory authorities who check that the study is being carried out correctly. The Trial Coordinating Centre may want to collect or copy some trial documents which will have your name and will include the signed Consent Form. This will help them to ensure that the trial is being carried out correctly. Your details will remain confidential and will be held in secure storage at the Trial Coordinating Centre. Your confidential information will be kept separately from the trial data and will be destroyed within five years of the trial ending. We will publish the results of the study in a medical journal so that other doctors can benefit from the knowledge, but your personal information will NOT be included and there will be no way that you can be identified.

**10) Can you change your mind about being in the study?**

You can always withdraw from the study at any time. You just need to say for example *“I’ve decided I don’t want to be in this study now”*. We hope that you will let us use information about how you got on, but if you do not want us to use it please tell the doctor.

**11) What else do you need to know?**

- In the event that something does go wrong and you are harmed during the study, the London School of Hygiene & Tropical Medicine who are organising the study would be responsible for claims for any non-negligent harm suffered as a result of participating in this study.
- We will ask you to sign a separate consent form and give you a copy to keep and you can also keep this information sheet.
- This study has been reviewed and approved by a Research Ethics Committee.

**12) What happens afterwards?**

If after you leave this hospital you develop any problems at any time up to 42 days after you had your baby, we would definitely want to know about it. You will be given a card with the contact details of the research doctor at this hospital, which you should keep safely and show to anyone who may be treating you for any illness.

If you would like to have a copy of the final results of this study, please let the research doctor know and s/he will ensure you receive a copy when it is published.
