# Supplementary material for: Effects of tranexamic acid on platelet function and thrombin generation (ETAPlaT): WOMAN trial sub-study
Source: Wellcome Open Res. 2016 Dec 15;1:29. [Version 1] doi: 10.12688/wellcomeopenres.9964.1 (PMC5234699; doi:10.12688/wellcomeopenres.9964.1)
Supplement: Supplementary file 2 [file wellcomeopenres-1-10739-s0001.tgz › 16c963f8-50e0-4579-b76d-5f1ec471f8e2.docx]

Patient consent form (Albanian)

Dr. Kastriot Dallaku, Spitali Universitar Obstetrik Gjinekologjik Koço Gliozheni,

Blv. Bajram Curri, Tirane, Albania Tel. +355 6920 54212, Email: kastriotdallaku@yahoo.com

FORMULARI I MIRATIMIT PËR PACIENTEN

**WOMAN trial and WOMAN-ETAPlaT (Studimi PËR FEMRAT)**

**TitulliStudimit**: (1) Acidi traneksamik për trajtimin e hemorragjisë së paslindjes: Një studim ndërkombëtar, i randomisuar, dyfish i verber, placebo dhe i kontrolluar. (2) WOMAN-ETAPlaT Efekti i Acidit Tranexamik ne funksionin trombocitar dhe gjenerimin e trombines, ne nje pjese te pacienteve te Studimit per FEMRAT.

| Numri i kodit të spitalit | 001 | Emri Investigatorit Kryesor lokal | | Dr.Kastriot Dallaku | | | | | |
| --- | --- | --- | --- | --- | --- | --- | --- | --- | --- |
| Numri identifikimit të spitalit të pacientes |  | | Numri randomisimit |  |  |  |  |  |  |
|  |  |  |  | KUTI | | | | PAKO | |
| Emriipacientes |  | | | | | | | | |

**Versioni Numër: 1.1 / Data e versionit: 3 qershor 2013 ju lutemi të vini inicialet në kuti**

1. Unë konfirmoj që e kam lexuar dhe e kuptoj fletushkën me informata, Versioni Numër 1, data e versionit 3 qershor 2013 për studimin e lartpërmendur dhe kam pasur mundësi të bëj pyetje.
2. Unë e kuptoj që pjesëmarrja ime është në mënyrë vullnetare dhe se jam e lirë të heq dorë në çfarëdo lloj kohe, pa dhënë ndonjë arsye dhe kështu pa patur ndikim në kujdesin tim shëndetësor dhe as në të drejtat e mia ligjore.
3. Unë e kuptoj që individë përgjegjës që përfshihen në këtë studim mund të shikojnë pjesë të shënimeve të mia mjekësore dhe të bebes/bebeve të mia. Unë i jap leje këtyre individëve që t’i shikojnë këto regjistrime.
4. Unë jap leje që një kopje e këtij formulari miratimi, që përmban të dhënat e mia personale, të vihet në dispozicion të Qendrës së Koordinimit të Eksperimentit në Londër vetëm për qëllime vëzhgimi.
5. Unë jap leje që mjeku im personal të marrë informata në lidhje me pjesëmarrjen time në këtë eksperiment.
6. Unë jam dakord të marr pjesë në studimin e lartpërmendur, studimi për FEMRAT (WOMAN trial dhe WOMAN-ETAPlaT).

_______________________________ ________________ ________________________________

Emri i pacientes Data Nënshkrimi / Shenja e gishtit të madh ose

shenjë tjetër (nëse nuk mund të nënshkruajë)

_______________________________ ________________ ________________________________

Emri i personit që merr miratimin Data Nënshkrimi

_______________________________ ________________ ________________________________

Emri investigatorit Kryesor lokal Data Nënshkrimi

*(Dëshmitari vetëm nëse është e nevojshme) Pacientja nuk është në gjendje të nënshkruajë dhe si dëshmitar unë konfirmoj që pacientes i janë dhënë të gjitha informatat për këtë eksperiment dhe ajo ka dhënë miratimin e saj me fjalë për të marrë pjesë.*

_______________________________ ________________ ________________________________

Emri i dëshmitarit Data Nënshkrimi

***Origjinali të mbahet në dosjen e studimit të hulumtuesit, 1 kopje për pacienten, 1 kopje të mbahet në regjistrimet e spitalit për pacienten.***

## Patient Consent Form (English)

Dr. Kastriot Dallaku, Obstetric Gynecology University Hospital Koço Gliozheni,

Blv. Bajram Curri, Tirane, Albania: Tel. +355 6920 54212, Email: kastriotdallaku@yahoo.com

CONSENT FORM FOR THE PATIENT

**THE WOMAN trial and WOMAN-ETAPlaT**

**Title of Research**: 1. Tranexamic acid for the treatment of postpartum haemorrhage: An international randomised, double blind, placebo controlled trial. 2. WOMAN-ETAPlaT – Effect of Tranexamic Acid on Platelet Function and Thrombin Generation, in a sample of participants of WOMAN trial

| Hospital Code Number | 001 | Name of Local Principal Investigator | | Dr. Kastriot Dallaku | | | | | |
| --- | --- | --- | --- | --- | --- | --- | --- | --- | --- |
| Patient Hospital ID Number |  | | Randomisation Number |  |  |  |  |  |  |
|  |  |  |  | BOX | | | | PACK | |
| Name of Patient |  | | | | | | | | |

**Version Number: 1.1 / Version Date: 3 June, 2013 Please initial boxes**

1. I confirm that I have read and understood the information sheet Version Number 1.1, version date 3 June, 2013, for the above study and have had the opportunity to ask questions.
2. I understand that my participation is voluntary and that I am free to withdraw at any time, without giving any reason and without my medical care or legal rights being affected.
3. I understand that sections of my medical notes and those of my baby/ies may be looked at by responsible individuals involved in the study. I give permission for these individuals to have access to these records.
4. I give permission for a copy of this consent form, which contains my personal information, to be made available to the Trial Coordinating Centre in London for monitoring purposes only.
5. I give permission for my personal doctor to be given information about my participation in this trial.
6. I agree to take part in the above study, the WOMAN trial and WOMAN-ETAPlaT.

_______________________________ ________________ ________________________________

Name of Patient Date Signature / Thumbprint

or other mark (if unable to sign)

_______________________________ ________________ ________________________________

Name of person taking consent Date Signature

_______________________________ ________________ ________________________________

Name of local principal investigator Date Signature

*(Witness only if required) The patient is unable to sign and as a witness I confirm that the patient has been given all the information about the trial and has verbally consented to taking part.*

_______________________________ ________________ ________________________________

Name of witness Date Signature

***Original to be filed in the Investigator’s Study File, 1 copy for patient, 1 copy to be kept with woman’s hospital records***
