# Supplementary material for: Effects of tranexamic acid on platelet function and thrombin generation (ETAPlaT): WOMAN trial sub-study
Source: Wellcome Open Res. 2016 Dec 15;1:29. [Version 1] doi: 10.12688/wellcomeopenres.9964.1 (PMC5234699; doi:10.12688/wellcomeopenres.9964.1)
Supplement: Supplementary file 3 [file wellcomeopenres-1-10739-s0002.tgz › 93caf845-6e38-419f-a5de-75ff6581638a.docx]

## Representative consent form (Albanian)

Dr. Kastriot Dallaku, Spitali Universitar Obstetrik Gjinekologjik Koço Gliozheni,

Blv. Bajram Curri, Tirane, Albania. Tel. +355 6920 54212, Email: kastriotdallaku@yahoo.com

FORMULARI I MIRATIMIT PËR PËRFAQËSUESIN E PACIENTES

**STUDIMI PËR FEMRAT**

**Titulliistudimit**: 1) Acidi traneksamik për trajtimin e hemorragjisë së paslindjes: Një studim ndërkombëtar, i randomisuar, dyfish i verber, placebo dhe i kontrolluar. (2) WOMAN-ETAPlaT – efkti i Acidit Tranexamik ne funksionin trombocitar dhe gjenerimin e trombines, ne nje pjese te paciente vete Studimit per FEMRAT.

| Numri i kodit të spitalit | 001 | Emri investiguesit kryesor lokal | | Dr.KastriotDallaku | | | | | |
| --- | --- | --- | --- | --- | --- | --- | --- | --- | --- |
| Numri i identifikimit të spitalit të pacientes |  | | NumriRandomisimit |  |  |  |  |  |  |
|  |  |  |  | KUTI | | | | PAKO | |
| Emri i pacientes |  | | | | | | | | |
| Emri i përfaqësuesit |  | | Lidhja e përfaqësuesit me pacienten |  | | | | | |

**Versioni Numër: 1.1 / Data e versionit: 3 qershor 2013 ju lutemi të vini inicialet në kuti**

Unë konfirmoj që e kam lexuar dhe e kuptoj fletën informative, Versioni Numër 1.1, data e versionit 3 qershor 2013, për studimin e lartpërmendur dhe kam pasur mundësi të bëj pyetje.

Unë konfirmoj që nuk di ndonjë arsye se përse kjo paciente do të kishte kundërshtuar të merrte pjesë në këtë studim.

Unë e kuptoj që pjesëmarrja ime është në mënyrë vullnetare dhe se jam i/e lirë të heq dorë në çfarëdo lloj kohe, pa dhënë ndonjë arsye dhe pa patur ndikim në kujdesin shëndetësor të pacientes dhe as në të drejtat e saj ligjore.

Unë e kuptoj që individë përgjegjës që përfshihen në këtë studim mund të shikojnë pjesë të shënimeve mjekësore të pacientes dhe të bebes/bebeve të saj. Unë i jap leje këtyre individëve që t’i shikojnë këto shënime.

Unë jap leje që një kopje e këtij formulari miratimi, që përmban të dhënat e mia personale, të vihet në dispozicion të Qendrës së Koordinimit të Eksperimentit në Londër vetëm për qëllime vëzhgimi.

Unë jap leje që mjeku personal i pacientes të marrë informacione në lidhje me pjesëmarrjen e saj në këtë eksperiment.

Unë jam dakord që pacientja e lart-përmendur të marrë pjesë në studimin për FEMRAT (WOMAN trial dhe WOMAN-ETAPlaT ).

__________________________________________________ ________________________

Nënshkrimi / shenja e gishtit të madh ose shenjë tjetër e Data

përfaqësuesit (nëse nuk mund të nënshkruajë)

_______________________________ ________________ ________________________________

Emri i personit që merr miratimin Data Nënshkrimi

_______________________________ ________________ ________________________________

Emri i investiguesit kryesor lokal Data Nënshkrimi

*(Dëshmitari vetëm nëse është e nevojshme) Përfaqësuesi nuk është në gjendje të nënshkruajë dhe si dëshmitar unë konfirmoj që përfaqësuesit i janë dhënë të gjitha informatat për këtë eksperiment dhe ai/ajo ka dhënë miratimin e tij/ saj me fjalë për të marrë pjesë.*

_______________________________ ________________ ________________________________

Emri i dëshmitarit Data Nënshkrimi

***Origjinali të mbahet në dosjen e studimit të hulumtuesit, 1 kopje për përfaqësuesin, 1 kopje të mbahet në dokumentet e spitalit për pacienten.***

Representative Consent Form (English)

Dr. Kastriot Dallaku, Obstetric Gynecology University Hospital Koço Gliozheni,

Blv. Bajram Curri, Tirane, Albania; Tel. +355 6920 54212, Email: kastriotdallaku@yahoo.com

CONSENT FORM FOR THE PATIENT’S REPRESENTATIVE

**THE WOMAN trial and WOMAN-ETAPlaT**

**Title of Research**: 1. Tranexamic acid for the treatment of postpartum haemorrhage: An international randomised, double blind, placebo controlled trial. 2. WOMAN-ETAPlaT – Effect of Tranexamic Acid on Platelet Function and Thrombin Generation, in a sample of participants of WOMAN trial

| Hospital code number | 001 | | Name of Local Principal Investigator | | Dr. Kastriot Dallaku | | | | | | | |
| --- | --- | --- | --- | --- | --- | --- | --- | --- | --- | --- | --- | --- |
| Patient Hospital ID Number | |  | | Randomisation Number | |  | |  |  |  |  |  |
|  |  |  |  |  |  | BOX | | | | | PACK | |
| Name of Patient |  | | | | | | | | | | | |
| Name of Representative |  | | | Relationship of representative to patient | | |  | | | | | |

**Version Number: 1.0 / Version Date: 03 June 2013 Please initial boxes**

1. I confirm that I have read and understood the information sheet Version Number 1, version date 03 June 2013, for the above study and have had the opportunity to ask questions.
2. I confirm that I am not aware of any reason why this patient would have objected to taking part in this study.
3. I understand that my consent is voluntary and that I am free to withdraw it at any time without giving any reason and without the patient’s medical care or legal rights being affected.
4. I understand that sections of the patient’s medical notes and those of her baby/ies may be looked at by responsible individuals involved in the study.
5. I give permission for a copy of this consent form which contains my personal information to be made available to the Trial Coordinating Centre in London for monitoring purposes only.
6. I give permission for the patient’s personal doctor to be given information about her participation in this trial.
7. I agree for the above named patient to take part in the WOMAN trial and WOMAN-ETAPlaT.

__________________________________________________ ________________________

Signature / thumbprint or other mark of Representative Date

(if unable to sign)

_______________________________ ________________ ________________________________

Name of person taking consent Date Signature

_______________________________ ________________ ________________________________

Name of local principal investigator Date Signature

*(Witness only if required) The representative is unable to sign and as a witness I confirm that the representative has been given all the information about the trial and has verbally consented to taking part.*

_______________________________ ________________ ________________________________

Name of witness Date Signature

***Original to be filed in the Investigator’s Study File, 1 copy for representative, 1 copy to be kept with woman’s hospital records***
