# Supplementary material for: Effects of tranexamic acid on platelet function and thrombin generation (ETAPlaT): WOMAN trial sub-study
Source: Wellcome Open Res. 2016 Dec 15;1:29. [Version 1] doi: 10.12688/wellcomeopenres.9964.1 (PMC5234699; doi:10.12688/wellcomeopenres.9964.1)
Supplement: Supplementary file 4 [file wellcomeopenres-1-10739-s0003.tgz › c32bd8ad-ecde-4504-8182-dc70ca788cfd.docx]

WOMAN-ETAPlaT data collection form – page 1

| 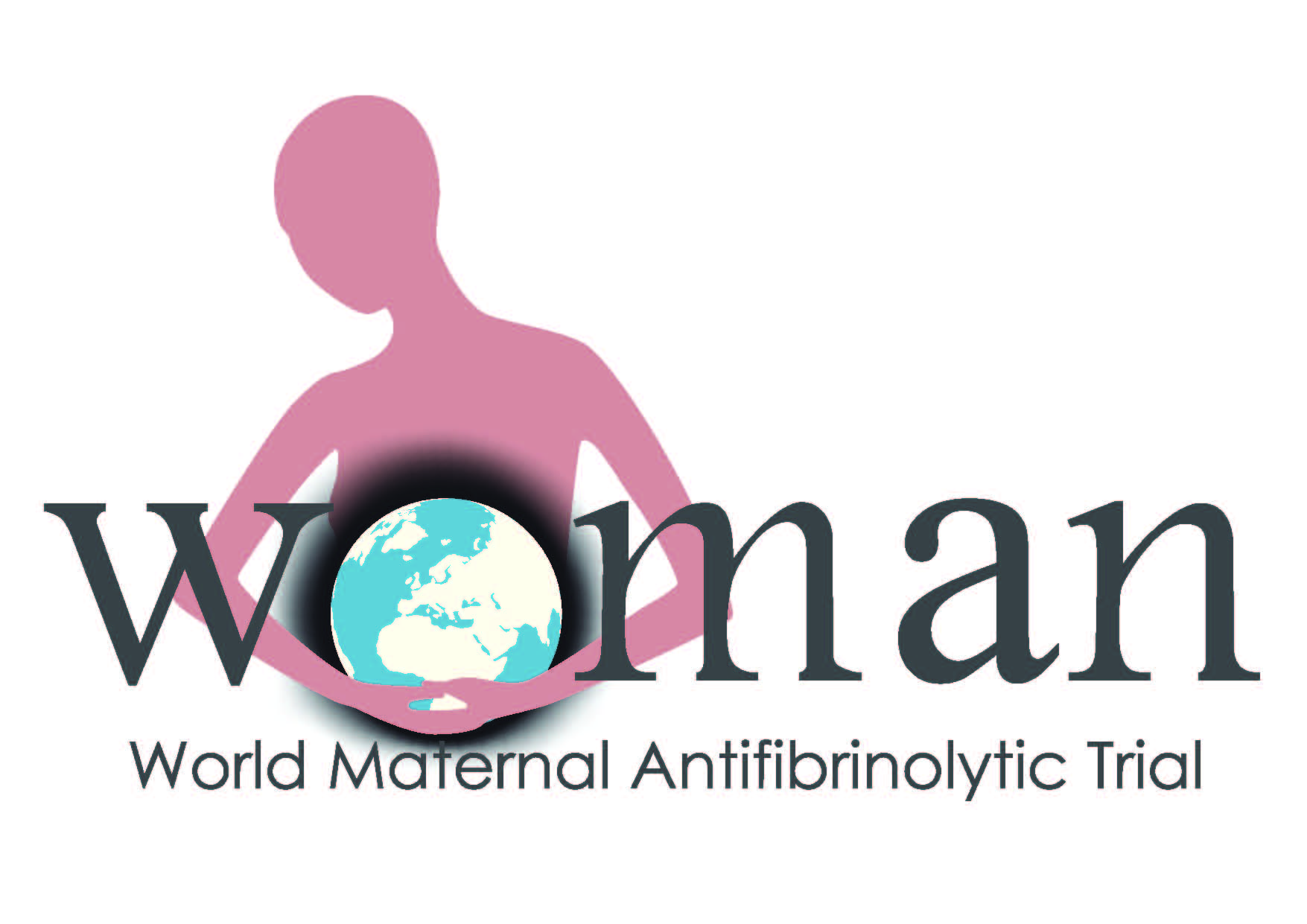 | WOMAN–ETAPlaT DATA COLLECTION FORM  The effect of TXA on platelet function and thrombin generation  in patients randomised to the WOMAN trial. |
| --- | --- |
|  | **PLEASE ENSURE ALL RELEVANT INFORMATION BELOW IS CONTAINED IN THE MEDICAL RECORDS.** |

**BASELINE DATA**

| 1. Hospital code |  | | | | 2. Patient’s initials (first name / last name) | | | |  |  |
| --- | --- | --- | --- | --- | --- | --- | --- | --- | --- | --- |
| 3.1 Parity *(circle one)* | | | | | | | | | NULIPARA | MULTIPARA |
| 3.2.1 Maternal height | meters | | | | 3.2.2 Maternal weight | | | | Kg | |
| 3.3 Gestational Age at birth | | | | | | | | | weeks | |
| 3.4 Birth Weight (BW) of fetus | | grams | | | | 2^nd^ fetus if twins | | | grams | |
| 3.5 Any concomitant diseases of pregnancy?  *(circle YES or NO on each line)* | | I | Preeclampsia | | | | | | YES | NO |
|  |  | Ii | Infection (chorioamnionitis) | | | | | | YES | NO |
|  |  | Iii | Diabetes | | | | | | YES | NO |
|  |  | Iv | Placental abruption | | | | | | YES | NO |
|  |  | V | Other (if yes, describe below) | | | | | | YES | NO |
| 3.5.1 Describe 3.5v *(if yes)*: | | | | | | | | | | |
| 3.6 Any pre-existing maternal disease?  *(circle YES or NO on each line)* | | I | Anaemia | | | | | | YES | NO |
|  |  | Ii | Any cardiac disease | | | | | | YES | NO |
|  |  | Iii | Renal disease | | | | | | YES | NO |
|  |  | Iv | Current treatment with antithrombotic drugs | | | | | | YES | NO |
|  |  | V | History of previous thromboembolism | | | | | | YES | NO |
|  |  | Vi | Any haemoglobinopathy | | | | | | YES | NO |
|  |  | Vii | Previous PPH | | | | | | YES | NO |
|  |  | viii | Other (if yes, describe below) | | | | | | YES | NO |
| 3.6.1 Describe 3.6vii *(if yes*): | | | | | | | | | | |
| 3.7 Use of anesthesia *(circle one answer)* | | | | GENERAL | | | SPINAL | | EPIDURAL | NONE |
| 3.8 Additional doses of uterotonics given? *(circle)* | | | | | | | | | YES | NO |
| 3.9 Labour induction / augmentation *(circle)* | | | | | | | | | YES | NO |
| 3.10 Total duration of labour – 1^st^ (active phase), 2^nd^ and 3^rd^ stage of labour | | | | | | | | 1^st^ (HH) | 2^nd^ (MM) | 3^rd^ (MM) |

| **4. Eligible for WOMAN trial** | **YES** | **NO** | 5. Time of venous blood sample | | | HH | MM |
| --- | --- | --- | --- | --- | --- | --- | --- |
| 6. Routine lab samples prepared for analyses *(circle)* | YES | NO | **NOTE:** Results to be printed off and both electronic output and pdf to be transferred to TCC. | | | | |
| 7. Any treatment which can affect Platelet Function (PF), TGA, Fibrinogen, D-Dimer, FV and FVIII levels *(circle)* | | | YES | NO | eg heparin, aspirin,  vitamin K, protamin | | |

**RANDOMISATION AND FIRST TREATMENT INFORMATION**

**Please enter on the form as processes are completed**

| 8. Insert randomisation number here | **BOX** |  |  |  |  | **PACK** |  |  |
| --- | --- | --- | --- | --- | --- | --- | --- | --- |

| 9. Date of randomisation | DD | MM | YYYY | 12.Time of Randomisation | HH | MM |
| --- | --- | --- | --- | --- | --- | --- |
| 10. Time first dose given | | | | | HH | MM |

## WOMAN-ETAPlaT data collection form – page 2

**POST TREATMENT DATA**

**Please complete immediately after second blood sample is collected.**

| 11. Time of post treatment venous blood sample | | HH | MM | 12. Second dose given | | | | | YES | NO |
| --- | --- | --- | --- | --- | --- | --- | --- | --- | --- | --- |
| 13. Any treatment which can affect PF, TGA, Fibrinogen, D-Dimer, FV & FVIII, given between samples 1 and 2? | | | | YES | NO | | eg heparin, aspirin, vit. K, protamine | | | |
| 14. Any Adverse Event directly associated with the  WOMAN-ETAPlaT Study? | | | | YES | NO | | If yes, report using WOMAN trial Adverse Event reporting procedure | | | |
| 15. Any technical problem with sampling or analysis of venous blood? | | | | YES | NO | | If yes describe in box 15.1 below | | | |
| 15.1 Describe 15 *(if yes)*: | | | | | | | | | | |
| 16. Name of person completing this data form |  | | | | | 17. Date | |  | | |
| 18. Signature |  | | | | | | | | | |

**WOMAN-ETAPlaT data collection form – page 3**

***The following data will be collected directly as electronic output from the test analysers for upload to the database. This is to show the data fields which will be collected. NO MANUAL ENTRY OF DATA REQUIRED.***

**BASELINE RESULTS OF ANALYSES Please print off pdf and electronic file output and transfer to the TCC.**

| 19. Multiplate® analyser | i | Date of analysis | DD | MM | | YYYY | Time of analysis | HH | MM |
| --- | --- | --- | --- | --- | --- | --- | --- | --- | --- |
|  |  | Multiplate test | Area Under Curve (U) | | Aggregation (AU) | | | Velocity (AU/min) | |
|  | ii | ADP test |  | |  | | |  | |
|  | iii | TRAP test |  | |  | | |  | |
| 20. Thrombin Generation Assay (TGA) | i | Date of analysis | DD | MM | | YYYY | Time of analysis | HH | MM |
|  | ii | Lag Time |  | | | | | min | |
|  | iii | Time to peak |  | | | | | min | |
|  | iv | Peak |  | | | | | nM | |
|  | v | ETP |  | | | | | nM*min | |
| 21. Coagulation parameters | i | Date of analysis | DD | MM | | YYYY | Time of analysis | HH | MM |
|  | ii | Fibrinogen |  | | | | | mg/dL | |
|  | iii | D-Dimer |  | | | | | mg/L | |
|  | iv | Coagulation Factor V |  | | | | | % | |
|  | v | Coagulation Factor VIII |  | | | | | % | |
|  | vi | Coagulation Factor vW |  | | | | | % | |

**FOLLOW-UP RESULTS OF ANALYSES Please print off pdf and electronic file output and transfer to TCC.**

| 22. Multiplate® analyser | i | Date of analysis | DD | MM | | YYYY | Time of analysis | HH | MM |
| --- | --- | --- | --- | --- | --- | --- | --- | --- | --- |
|  |  | Multiplate test | Area Under Curve (U) | | Aggregation (AU) | | | Velocity (AU/min) | |
|  | ii | ADP test |  | |  | | |  | |
|  | iii | TRAP test |  | |  | | |  | |
| 23. Thrombin Generation Assay (TGA) | i | Date of analysis | DD | MM | | YYYY | Time of analysis | HH | MM |
|  | ii | Lag Time |  | | | | | min | |
|  | iii | Time to peak |  | | | | | min | |
|  | iv | Peak |  | | | | | nM | |
|  | v | ETP |  | | | | | nM*min | |
| 24. Coagulation parameters | i | Date of analysis | DD | MM | | YYYY | Time of analysis | HH | MM |
|  | ii | Fibrinogen |  | | | | | mg/dL | |
|  | iii | D-Dimer |  | | | | | mg/L | |
|  | iv | Coagulation Factor V |  | | | | | % | |
|  | v | Coagulation Factor VIII |  | | | | | % | |
|  | vi | Coagulation Factor vW |  | | | | | % | |

**ROUTINE LABORATORY PARAMETERS Please print off pdf and electronic file output and transfer to TCC.**

|  | **(a) Before delivery** | | | | | **(b) After delivery** | | | | |
| --- | --- | --- | --- | --- | --- | --- | --- | --- | --- | --- |
| 25. Date of analysis | DD | MM | | YYYY | | DD | MM | | YYYY | |
| 26. Time of analysis | HH | | MM | | | HH | | MM | | |
| 27. White blood cells (Leu) |  | | | | 10^3^/μL |  | | | | 10^3^/μL |
| 28. Red blood cells (Er) |  | | | | 10^6^/μL |  | | | | 10^6^/μL |
| 29. Haemoglobine (Hb) |  | | | | g/dL |  | | | | g/dL |
| 30. Haematocrit (Hct) |  | | | | % |  | | | | % |
| 31. Platelets (Plt) |  | | | | 10^3^/μL |  | | | | 10^3^/μL |
| 32. Mean Platelet Volume (MPV) |  | | | | μm^3^ |  | | | | μm^3^ |
