# Supplementary material for: Effects of tranexamic acid on platelet function and thrombin generation (ETAPlaT): WOMAN trial sub-study
Source: Wellcome Open Res. 2016 Dec 15;1:29. [Version 1] doi: 10.12688/wellcomeopenres.9964.1 (PMC5234699; doi:10.12688/wellcomeopenres.9964.1)
Supplement: Peer review history

# Peer review: Referee response for version 1

**DOI:** 10.21956/wellcomeopenres.10739.r18542

**Published:** 2017-01-11

**Authors:** Loïc Sentilhes

**Affiliations:** 1 Department of Obstetrics and Gynecology, Bordeaux University Hospital, Bordeaux, France

The authors have published their protocol of a WOMAN trial sub-study.

 WOMAN is a pragmatic, randomized, double-blinded, placebo-controlled trial in women with a clinical diagnosis of postpartum hemorrhage (PPH) and is intended to determine reliably the effect of the early administration of TXA on death, hysterectomy, and other morbidity (surgical intervention, blood transfusion and risk of nonfatal vascular events). Recruitment of the 20,000 planned randomized women has been completed as well as statistical analysis.

 The aim of this sub-study is to assess the effect of TXA on thrombin generation but also on platelet function, fibrinogen, D-dimer and coagulation factors in women with PPH. The authors hypothesize that TXA will decrease thrombin generation by inhibiting plasmin and modify platelet activity, coagulation factors V and VIII and Von Willebrand levels in women with PPH. The protocol of the WOMAN trial has been previously published elsewhere.

 In this sub-study, it is planned, only on a single site located in Albania, to obtain blood samples immediately after randomization and before the administration of the first dose of trial treatment as well as about 30±15 minutes after the administration of the first dose of trial drugs (but before the second dose of trial treatment if required).

 Platelet function will be assessed immediately on site using Multiplate®tests, while other samples will be centrifugated and preserved at -80°C to be transferred in Germany for later analysis of Thrombin generation Assay, fibrinogen, D-dimers, coagulation factors V and VIII and Von Willebrand levels.

 The protocol is well written, original and will provide useful, interesting and likely robust data related to the impact of TXA on hemostasis of PPH women. I have only minor comments.

- The authors should explain why only one site participates to the WOMAN trial sub-study.
- The primary outcome should be clinically (and in pathophysiological manner) justified: Endogenous Thrombin Potential difference of 243nM/min.  
- The sample size should be based on literature or the authors should mention that, as this analysis is exploratory, it is somewhat arbitrary.
- The authors should clearly note that analysis will be performed blindly.
- The authors should clearly mention what material laboratory will be used to assess Thrombin generation Assay, fibrinogen, D-dimers, coagulation factors V and VIII and Von Willebrand levels.

I have read this submission. I believe that I have an appropriate level of expertise to confirm that it is of an acceptable scientific standard.

# Peer review: Referee response for version 1

**DOI:** 10.21956/wellcomeopenres.10739.r18827

**Published:** 2017-01-03

**Authors:** Anne-Sophie Ducloy-Bouthors, Emmanuelle Jeanpierre

**Affiliations:** 1 Pôle d'Anesthésie-Réanimation, Hôpital Jeanne-de-Flandre, CHRU Lille, Lille, France; 2 Department of Hematology and Transfusion, Centre de Biology Pathology, Centre Hospitalier Régional, Lille, France

The authors plan to conduct a sub-study within the WOMAN trial. Among the women experiencing clinically diagnosed primary PPH after vaginal or caesarean delivery and included in the WOMAN trial, blood samples will be collected at baseline and 30 minutes after the first dose of study treatment. Using platelet poor plasma, thrombin generation, fibrinogen, D-dimer, factor V and VIII, and Von Willebrand factor will be measured. Platelet function will be evaluated in whole blood using Multiplate® tests. The primary outcome is the effect of TXA on thrombin generation. Secondary outcomes include the effect of TXA on platelet function, fibrinogen, D-dimer and coagulation factors.

 The title is appropriate for the content of the article and the abstract represent a suitable summary of the work. The trial is well described regarding the background, the method and the expected results. The conclusions are sensible, balanced and justified on the basis of the results of the study.This paper is of interest to explore the hypothesis of TA-related thrombin generation inhibition and I recommend indexing it with minor corrections.

- One suggestion and the aim of our TRACES trial [CT 02797119] should be to analyze simultaneously the plasmin inhibition to link the force of the thrombin procoagulant effect and plasmin TA inhibition.
- Please clarify which laboratory will perform the biological tests and the expected delay between the samples and the LAB analysis.
- Please clarify if the biological data analysis will be blinded to the allocation?

I believe the data is in a usable format/structure and there is sufficient information provided for the experiment to be replicated.

We have read this submission. We believe that we have an appropriate level of expertise to confirm that it is of an acceptable scientific standard.
